# How to Teach Healthy Life-Style Efficiently in a Pediatric Outpatient Setting: Proposal of an Innovative Tridimensional Pyramid

**DOI:** 10.3390/nu18081209

**Published:** 2026-04-11

**Authors:** Angelika Anna Mohn, Giada Di Pietro, Alessandro Maggitti, Giulia Trisi, Ilaria Bucci, Martina Passarelli, Nella Polidori, Armando Di Ludovico, Francesco Chiarelli

**Affiliations:** 1 Departement of Pediatrics, University of Chieti, 65100 Chieti, Italy; giada.dipietro@studenti.unich.it (G.D.P.); alessandro.maggitti97@gmail.com (A.M.); giuliatrisi.95@outlook.it (G.T.); ilariabbi3@gmail.com (I.B.); martinapassarelli96@gmail.com (M.P.); nella.polidori@hotmail.it (N.P.); armandodl@outlook.com (A.D.L.); chiarelli@unich.it (F.C.); 2 Clinical Research Center, “G.d’Annunzio” Foundation, University of Chieti, 65100 Chieti, Italy

**Keywords:** pediatric obesity, lifestyle education, Mediterranean diet, physical activity, sleep hygiene, health promotion, tridimensional pyramid

## Abstract

Background: Childhood obesity is a major public health concern associated with adverse metabolic outcomes later in life. Despite increased awareness, unhealthy lifestyle behaviors—including suboptimal diet quality, physical inactivity, insufficient sleep, and unfavorable body composition—remain prevalent in pediatric populations. Effective, child-centered educational tools for early prevention are still limited. Methods: We developed the Lifestyle Tridimensional Pyramid, an educational model integrating nutrition, physical activity, and sleep within a single, three-dimensional framework. The model also addresses body composition by emphasizing the balance between skeletal muscle and adipose tissue and the interdependence of lifestyle behaviors. This narrative review is supported by an umbrella review of 17 systematic reviews and meta-analyses published between 2010 and 2025, synthesizing evidence on lifestyle behaviors of pediatric obesity. Results: High- to moderate-quality evidence indicates that adherence to Mediterranean-style dietary patterns, regular moderate-to-vigorous physical activity, adequate sleep duration, and a healthier body composition might prevent the development of obesity and improved cardiometabolic profiles in children and adolescents. The pyramid provides a structured, visually accessible tool to support lifestyle counseling in pediatric outpatient settings and is adaptable to school- and community-based health promotion. Conclusions: Although prospective validation studies are warranted, the Lifestyle Tridimensional Pyramid represents a practical, evidence-informed framework to support integrated lifestyle education and improve primary and secondary prevention of pediatric obesity.

## 1. Introduction: Why Is It Important to Teach Healthy Life-Style Starting in Childhood?

Childhood obesity represents an ongoing challenge for both primary and secondary prevention in order to avoid adipose tissue overgrowth and its associated complications [1]. The rising prevalence of pediatric obesity, which is expected to lead to a growing number of adults with long-term metabolic complications (such as type 2 diabetes, dyslipidemia, hypertension and cardiovascular disease), highlights the need for effective strategies to reverse this alarming trend [2]. In fact, we are already witnessing the detrimental impact of obesity on adult health, to the extent that it has finally been officially recognized as a disease. Italy is the first country in the world to legally acknowledge obesity as a “progressive and relapsing” condition.

On 1 October 2025, the Italian Senate definitively approved Bill No. 1483 (commonly referred to as the “Pella Law,” named after the Forza Italia MP who proposed and co-signed it), which addresses the prevention, treatment, and social inclusion of individuals living with obesity—currently estimated at around six million people in Italy (excluding those who are overweight) [3]. This landmark decision represents a turning point, not only reflecting growing awareness among the general public, policymakers, and the scientific community, but also laying the foundation for formally recognizing the opportunity to counter the obesity epidemic. It emphasizes the need for a comprehensive, preventive strategy—particularly during childhood, which constitutes the most sensitive and critical window for intervention [4]. Children and adolescents today are embedded within environments that inadequately support, and often actively undermine, healthy development. These obesogenic settings—characterized by pervasive exposure to energy-dense, nutrient-poor food options and limited opportunities for physical activity—promote sedentary behavior and social disengagement. Proximity to fast-food vendors near schools has been linked to higher BMI and body fat in pediatric populations [5]. Globally, the coexistence of obesogenic behaviors (e.g., poor dietary patterns, low physical activity, high sedentary time) remains highly prevalent among adolescents, with 9.8–10.2% concurrently exhibiting three or more such behaviors across multiple countries between 2003 and 2017 [6]. Additionally, a large-scale meta-analysis revealed that neighborhoods with better infrastructure—like bike lanes, sidewalks, and green spaces—are linked to lower childhood obesity rates [7]. The Mediterranean dietary pattern is widely acknowledged for its favorable impact on health outcomes. The Mediterranean diet, defined by Ancel Keys in the 1960s, is one of the most well-known and well-researched dietary patterns worldwide; it has been recognized by UNESCO as an “Intangible Cultural Heritage” that is deeply rooted into its geographical origin and whose agricultural and dietary practices have a responsible interaction with the environment [8].

The Mediterranean diet, traditionally rooted in regions of olive cultivation, has long been associated with a reduced risk of chronic non-communicable diseases—including cardiovascular disease, obesity, and metabolic disorders—and with increased life expectancy [9]. This dietary pattern is characterized by a high intake of plant-based foods such as fruits, vegetables, legumes, nuts, and minimally processed cereals, along with olive oil—particularly extra-virgin olive oil—as the primary source of monounsaturated fats [10]. It also includes moderate consumption of dairy products (mainly yogurt and cheese), as well as low-to-moderate intake of fish, poultry, and eggs, with limited consumption of red and processed meats [11].

Due to its high content of nutrient-dense and minimally processed foods, the Mediterranean diet represents a healthy and balanced dietary model suitable across the lifespan, including pediatric populations [12]. Furthermore, beyond its nutritional benefits, it is increasingly recognized as a sustainable dietary pattern, associated with lower environmental impact and aligned with current global priorities related to food security, climate change, and the promotion of both human and planetary health [13].

Nevertheless, in geographical regions where adherence would apparently be more attainable, such as southern Europe, the prevalence of childhood obesity continues to escalate [14]. In a cohort of children from Southern Italy, 71.2% showed poor adherence to the Mediterranean diet, 26.5% had moderate adherence, and only 2.3% demonstrated good adherence [15]. Moreover, obesity etiology extends beyond suboptimal dietary patterns to encompass a broader spectrum of unhealthy lifestyle behaviors—among them, physical inactivity, excessive screen time, and disrupted sleep patterns. These factors not only contribute to reduced adherence to the Mediterranean diet but also exacerbate obesity risk in children aged 5–12 years [16].

Given this context, there is a compelling need to strengthen preventive strategies through early-life health promotion in pediatric settings. Lifestyle interventions initiated in childhood can have prolonged metabolic benefits, as demonstrated by long-term alterations in biomarkers even years after the initial intervention period [17].

In summary, contemporary societal and environmental contexts create multiple, reinforcing pressures that foster unhealthy eating, sedentary lifestyles, and social isolation in young populations. Such obesogenic environments represent a significant barrier to pediatric health that demands multifaceted, systemic interventions at the individual and community levels.

## 2. Modern Paradox: Greater Knowledge, Worse Habits

Before addressing how to educate children and their parents to understand and adhere to a healthy lifestyle, it is important to reflect on how we have reached the current situation. Historically, knowledge of the complications of obesity was far more limited, yet the condition was considerably less prevalent. Today, despite substantial scientific evidence on the health consequences of obesity, its prevalence continues to rise globally, and healthcare professionals often appear to face twice the effort in educating patients and their families about the associated risks [18].

Knowledge alone, however, is insufficient. Unhealthy behaviors persist as a result of a complex interplay among environmental, biological, psychological, and social factors [19]. Effective interventions therefore require multidimensional strategies, including environmental and policy measures, education, psychological support, more accurate diagnostic approaches, and broad societal change.

As a matter of fact, BMI, waist circumference and waist circumference to hip ratio are often used as an index of anthropometric evaluation but may mask the true problem of an improper lifestyle, which will become evident with a pathological BMI when it may be too late [20].

One major barrier is the information overload surrounding diet and health, which is not always grounded in scientific evidence. The spread of dietary fads and misinformation contributes to confusion, making it difficult for the public to distinguish evidence-based recommendations from commercial messaging [21].

Modern society is characterized by an “obesogenic environment”, where the continuous availability of inexpensive, energy-dense, and highly palatable foods—rich in sugars, fats, and salt—facilitates unhealthy choices [22]. Food marketing, particularly when directed toward children, strongly influences dietary preferences, while urban lifestyles reduce time for meal preparation and favor reliance on fast foods and processed products [23]. NOVA defines “Ultraprocessed foods” as industrial formulations generated through compounds extracted, derived or synthesized from food or food substrates. Such consumables typically contain five or more ingredients per product while scarcely containing intact or unprocessed wholefood. Ultraprocessed foods also commonly contain artificial substances such as colours, sweeteners, flavours, preservatives, thickeners, emulsifiers and other additives used to promote aesthetics, enhance palatability and increase shelf life. Both the low nutritional quality and high-energy density profiles common to ultraprocessed food are widely accepted as critical drivers of chronic noncommunicable diseases [24].

Although the importance of physical activity is widely recognized, daily life has become increasingly sedentary, dominated by motorized transport, office-based occupations, and screen-based leisure [25]. Stressful routines and time constraints frequently drive individuals toward quick, immediately rewarding choices. Moreover, chronic stress, insufficient sleep, and sedentary work promote compensatory behaviors such as emotional eating [26]. Importantly, food is not only a source of nutrition but also of gratification, and changing deeply ingrained habits requires motivation, social support, and tailored strategies—mere awareness of “what is right” is not sufficient [27].

In response to these challenges, we have developed an updated version of the traditional food pyramid—now reconceptualized as a Lifestyle Tridimensional Pyramid tailored specifically for children and adolescents. To support this updated framework, we conducted an umbrella review of systematic reviews, providing a robust synthesis of the existing evidence that underpins the proposed model and is presented in the subsequent paragraph. Our model aims to integrate recommendations not only for dietary quality and eating behavior, but also for physical activity and sleep hygiene [28]. Furthermore, it illustrates the importance of a well balanced muscle-fat body composition in relation to protective and promotive factors on health [29]. It aims to enhance relevance, clarity, and accessibility, thereby empowering pediatric clinicians, educators, and caregivers to more effectively promote sustainable healthy lifestyle.

## 3. Umbrella Review on Pediatric Lifestyle

### 3.1. Methods

This umbrella review was conducted following PRISMA 2020 and the PRISMA-Umbrella extension [30]. Searches were conducted in PubMed/MEDLINE, Embase, and Cochrane Database of Systematic Reviews, supplemented by reference tracking. Only English-language publications were included. Titles and abstracts were independently screened by four reviewers, followed by full-text assessment, with discrepancies resolved through discussion. The study selection process followed PRISMA 2020 guidelines [30] and is illustrated in Supplementary Figure S1. A total of 450 records were identified, of which 387 remained after duplicate removal. To address potential overlap of primary studies across included reviews, we compared the study lists of the included reviews. Although a formal quantitative assessment was not performed, overlap was qualitatively evaluated and, when identified, findings were interpreted cautiously, prioritizing the most comprehensive and recent evidence. Following title and abstract screening, 75 articles underwent full-text assessment, and 17 systematic reviews and meta-analyses were ultimately included.

Eligibility criteria included systematic reviews and meta-analyses published in peer-reviewed journals between 2010 and 2025 that investigated associations between lifestyle-related factors (diet quality, physical activity, sleep, or body composition) and obesity or related metabolic outcomes in children and adolescents (0–18 years).

Exclusion criteria comprised narrative reviews without a systematic methodological approach, primary research studies, conference abstracts, study protocols, and unpublished reviews. Furthermore, studies exclusively involving adult populations or addressing pharmacological or surgical interventions were excluded.

Data on review characteristics, population age range, intervention/exposure, main outcomes, and key findings were extracted independently by four reviewers using a standardized form. Due to heterogeneity in exposures and outcomes, results were synthesized narratively across four lifestyle domains: diet quality, physical activity, sleep, and body composition. The methodological quality of included reviews was independently assessed by four reviewers using the AMSTAR-2 tool, with discrepancies resolved through discussion. Overall confidence was classified as high, moderate, low, or critically low. The certainty of evidence across lifestyle domains was then evaluated based on consistency of findings, methodological quality, and sample size, and categorized as high, moderate, or low.

### 3.2. Results

A total of 17 systematic reviews and meta-analyses were included (Table 1), most assessing BMI or adiposity, with fewer reporting metabolic biomarkers or detailed body composition. The methodological quality of included reviews varied, with most rated as moderate to high confidence according to AMSTAR-2.

Overall, high-certainty evidence indicates the importance of adherence to Mediterranean-style or generally healthy dietary patterns [16,22,31,32,33,34] and regular moderate-to-vigorous physical activity [7,35,36], while poor diet quality, high intake of ultra-processed foods, and sedentary behaviors [37,38] increase obesity and metabolic risk.

Regular moderate-to-vigorous physical activity improves BMI, insulin sensitivity, lipid profile, and overall cardiometabolic health in children and adolescents [7,25,35,36,39,40,41], independently of adiposity and partly mediated by skeletal muscle mass.

Moderate-to-high quality evidence highlights the importance of sufficient and good-quality sleep in weight regulation and metabolic health [32,37,42,43] whereas short sleep duration or poor sleep quality increases risk. Maintaining an adequate muscle-to-fat ratio is linked to improved cardiometabolic profiles even at similar levels of adiposity [38,44].

Importantly, reviews examining multi-component interventions consistently show that integrated adherence to healthy diet, regular physical activity, adequate sleep, and favorable body composition confers the lowest obesity and metabolic risk, supporting the need for holistic strategies over isolated behavior changes [7,44,45,46]. Overall, the certainty of evidence was high for physical activity and diet quality, moderate for sleep, and limited for body composition outcomes.

**Table 1:** Characteristics of Included Systematic Reviews and Meta-Analyses.

	Author (Year)	Study Type	Population (Age)	Exposure/Domain	Main Outcomes	Key Findings	AMSTAR- 2 Quality
[16]	Masini et al., 2024	Umbrella review	Children & adolescents	Mediterranean diet	BMI, adiposity, metabolic risk	Higher adherence associated with improved anthropometric and metabolic outcomes	High
[22]	Petridi et al., 2024	Systematic review	Children & adolescents	Ultra-processed foods	BMI, cardiometabolic risk	UPF intake positively associated with obesity	High
[25]	Christian et al., 2024	Systematic review	Young children	Physical activity	PA levels, sedentary time	Decline in PA with age; sedentary behavior increases risk	High
[30]	Larruy-Garcia et al., 2024	Systematic review & meta-analysis	Children & adolescents	Diet quality	Obesity, metabolic syndrome	Poor diet quality associated with higher obesity/metabolic risk	High
[31]	Wang et al., 2023	Meta-analysis	Children & adolescents	Breakfast consumption	Obesity risk	Breakfast skipping increases obesity risk	High
[32]	Zheng et al., 2023	Systematic review & meta-analysis	Children & adolescents	Diet quality	BMI, obesity, metabolic outcomes	Higher diet quality associated with lower BMI and reduced metabolic risk	High
[34]	Pozuelo-Carrascosa et al., 2018	Systematic review & meta-analysis	Children & adolescents	Physical activity (school-based)	Cardiometabolic risk factors	Regular school-based PA improves cardiometabolic risk indicators (BP, lipids, glucose, insulin)	High
[36]	Garg et al., 2025	Meta-analysis	Children	Screen eating	Energy intake	Eating while watching TV increases energy intake	High
[37]	Calcaterra et al., 2024	Systematic review	Children & adolescents	Muscle health	Insulin resistance	Muscle mass protective against metabolic risk	High
[38]	Jaeger et al., 2023	Systematic review	Children (3–8 y)	Meal timing/distribution	Energy intake	Improper meal timing linked to excess energy intake	Moderate
[39]	Berman et al., 2012	Narrative systematic review	Children & adolescents	Physical activity	Insulin sensitivity	PA improves insulin sensitivity independent of adiposity	Moderate
[40]	Liu et al., 2024	Systematic review & meta-analysis	Children & adolescents	Aerobic + resistance exercise	BMI, adiposity, body composition, metabolic biomarkers	Combined aerobic and resistance training improves BMI, body fat, waist circumference, and metabolic biomarkers	High
[41]	Grimaldi et al., 2023	Systematic review & meta-analysis	Children & adolescents	Sleep duration	BMI, adiposity	Short sleep associated with increased obesity risk	High
[42]	Cai et al., 2024	Systematic review & meta-analysis	Children & adolescents	Sleep disturbances/ Sleep quality	Prevalence of sleep disturbances	High prevalence (~34%) of sleep disturbances; associations with behavioral/mental health concerns	High
[43]	Albornoz-Guerrero et al., 2021	Systematic review	Children & adolescents	Diet & PA	Obesity risk	Healthy lifestyle (diet + PA) associated with lower obesity risk	High
[44]	Pearson et al., 2025	Systematic review & meta-analysis	Adolescents (10–19 y)	Combined diet + physical activity	Dietary behavior, PA, adiposity outcomes	Combined diet & PA interventions improve behaviors and adiposity outcomes in adolescents	High
[45]	Waters et al., 2011	Systematic review	Children & adolescents	Lifestyle (diet + PA + behavioral)	BMI, adiposity, obesity risk	Multicomponent lifestyle interventions reduce BMI and improve weight outcomes	High

## 4. The Lifestyle Tridimensional Pyramid

These findings provide a robust evidence base for the proposed pyramid. By visually integrating nutrition, physical activity, sleep, and body composition into a single, three-dimensional model, the pyramid reflects the interconnected nature of lifestyle behaviors in influencing pediatric health. This model was developed and systematically refined through iterative testing with families and patients over an extended period of nearly three years, ensuring its relevance, feasibility, and acceptability in real-world pediatric settings. The umbrella review data reinforce the emphasis on maintaining a healthy muscle-to-fat ratio and support educational approaches aimed at preventing obesity and promoting long-term metabolic health.

### 4.1. The Food Choice Triangle

**Figure 1:**
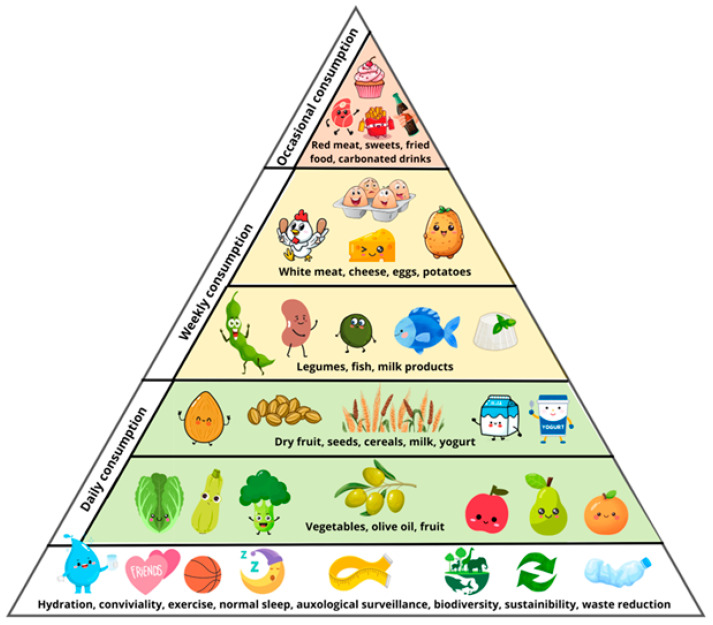
The 1st face: “The food choice triangle”.

The “Food Choice Triangle” (Figure 1) of our tridimensional pyramid represents the updated version of the Mediterranean Diet Pyramid proposed by the Italian Society of Human Nutrition (SINU) earlier this year, slightly adapted to pediatric outsetting [47]. This new model was developed as a tool to support educational activities and public health campaigns promoting a healthy and sustainable diet. Core values such as biodiversity, environmental sustainability, and waste reduction should be introduced early in life [48]. Pediatric outpatient visits offer a valuable opportunity to foster awareness and reflection on these topics among children and their families. The grounds of a healthy lifestyle need to be established during infancy and adolescence, emphasizing key elements such as family meals, adequate sleep patterns, and regular physical activity—particularly in the form of unstructured play and organized sports. It is essential to clearly communicate to both parents and children that age-appropriate sleep duration is not only a primary determinant of healthy cognitive development, longitudinal growth and timely pubertal progression but plays a central role in preventing excessive weight gain [42].

In this context, the focus in pediatrics should not be solely on weight control, but rather on routine auxological surveillance. This approach facilitates the early identification of abnormal growth trajectories that may signal an increased risk for the development of childhood obesity and represents a crucial window for early intervention [49]. Water should be promoted as the exclusive beverage of choice, without the addition of flavorings or sweeteners [50]. Therefore, the consumption of energy drinks among adolescents as well as juices among infants must be discouraged. The structure of the food choice pyramid—ranging from foods recommended for daily consumption to those intended for occasional intake—remains almost the same in the pediatric setting. However, in a pediatric context it is particularly important to promote nutritional education that raises awareness among children and families of the need to include vegetables daily in both lunch and dinner as a preventative measure against excess weight gain and future metabolic complications [51]. Furthermore, nutritional requirements change throughout childhood, and during puberty, it is particularly important to ensure sufficient milk consumption as a source of calcium, protein, and other nutrients critical for bone growth and overall development [52].

### 4.2. The Daily Food Distribution Triangle

**Figure 2:**
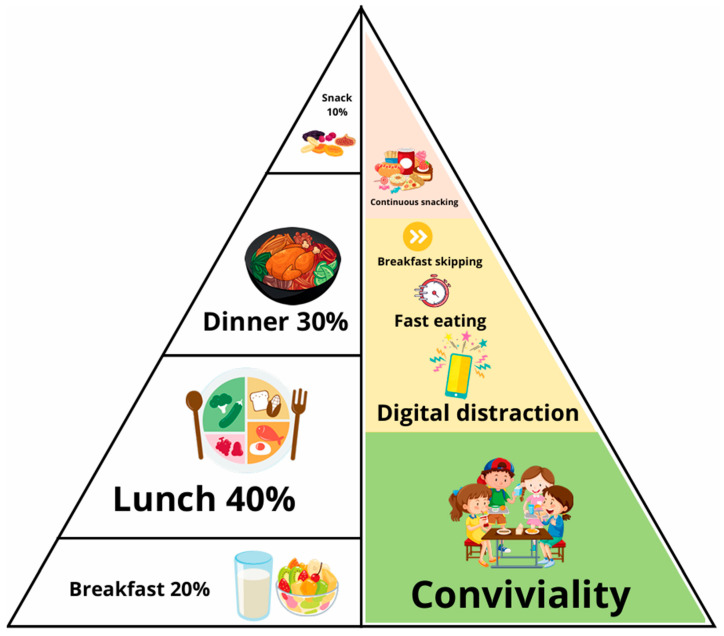
The 2nd face: “The daily food distribution triangle”.

This schematic representation (Figure 2) is divided into two sections: on the left, it illustrates the recommended daily meal distribution, while on the right, it highlights the most frequent dietary errors. The recommended dietary pattern consists of five meals per day—breakfast, lunch, dinner, and two snacks. Breakfast is positioned at the base of the diagram, symbolizing its role as the starting point of daily food intake. Notably, breakfast omission is among the most prevalent dietary mistakes [32]. Lunch constitutes the main caloric intake of the day, whereas breakfast and dinner combined should account for approximately 50% of total daily energy consumption. Snacks should contribute no more than 10% of daily caloric intake [39]. Children of kinder-garden age might need two snacks due to a physiologically shorter duration of fasting tolerance [53,54]. Frequent snacking and rapid eating are identified as key nutritional errors [55]. Persistent snacking interferes with the physiological postprandial suppression of insulin between meals, while rapid eating compromises the onset of satiety, potentially leading to overeating [56].

Furthermore, eating while distracted—such as during television viewing, phone use, or other forms of entertainment—reduces meal awareness and satiety perception [37]. One of the fundamental principles of healthy eating behavior is conviviality, which promotes shared meals, social interaction, and overall psychological well-being [57]. This visual tool enables patients to clearly recognize how improper meal timing and common eating behaviors contribute to an unhealthy lifestyle.

### 4.3. The Muscle–Adipose Tissue Triangle

**Figure 3:**
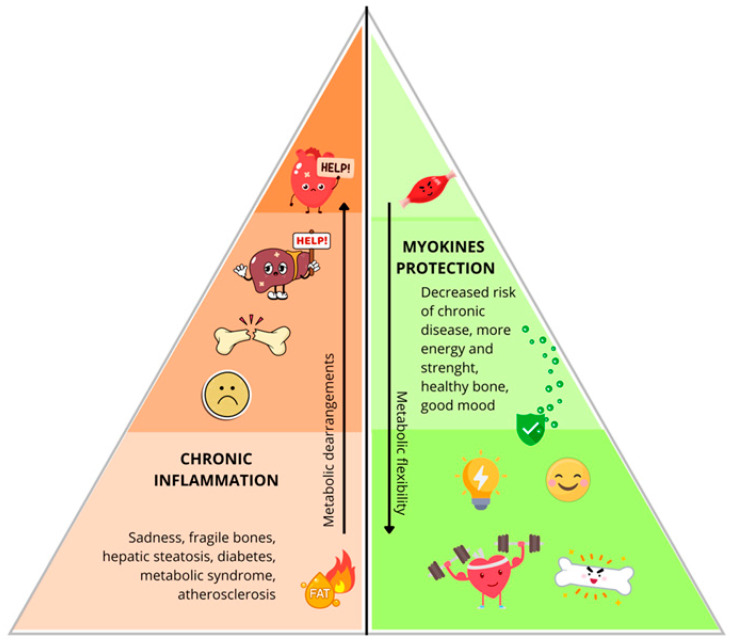
The 3rd face: “The muscle–adipose tissue triangle”.

This schematic figure (Figure 3) illustrates the balance between skeletal muscle and adipose tissue, two metabolically active compartments whose interaction plays a critical role in determining health trajectories from early life.

A bipartite color gradient is employed: on the right side, muscle tissue (green), varying from deep green at the base—indicating high muscle mass—to pale green at the apex—indicating low muscle mass; on the left, adipose tissue (red), moving from pale red at the base—minimal fat stores—to deep red at the apex—excessive adiposity.

The base of the figure is the physiological ideal state, characterised by high muscle mass and low fat mass. In fact in this physiological ideal state with low fat mass, adipose tissue consists of a large number of small healthy adipocytes and healthy immune cells which is combined with a immune-metabolic homeostatic state [58]. In contrast increasing adipose tissue mass leads to dysfunctional adipose tissue in obesity which perpetuates chronic low-grade inflammation via hypertrophic adipocytes and immune cell infiltration, thereby driving systemic insulin resistance and metabolic disease [59].

Skeletal muscle, now recognized as an endocrine organ, contributes to metabolic flexibility via myokine release; these myokines enhance glucose uptake, promote lipid oxidation, improve insulin sensitivity, and mitigate chronic low-grade inflammation [38].

In contrast, the apex of the image represents metabolic dysregulation, a state marked by low muscle mass together with excessive adiposity. Consequences include systemic insulin resistance, dyslipidemia, elevated blood pressure, and increased visceral fat deposition. These derangements are associated with elevated risk for obesity, type 2 diabetes, and cardiovascular disease. Childhood adiposity has been shown through Mendelian randomization to causally contribute to future adult coronary heart disease, myocardial infarction, heart failure, and atrial fibrillation [60].

Beyond these metabolic effects, adequate muscle mass correlates with reduced fatigue, increased physical energy, and improved mood regulation—factors especially important when educating children about healthy lifestyle habits. Excess adipose tissue is also frequently linked to diminished vitality and an increased predisposition to low mood, compounding the adverse health profile.

In children with overweight/obesity but preserved muscle strength, anthropometric and cardiovascular risk markers are lower than in those with low strength, even when fat mass is similar [44].

By juxtaposing these two extremes, the figure highlights the importance of maintaining an appropriate ratio of muscle to fat mass. This conceptual framework may serve as an effective pedagogical tool for children, emphasizing that regular physical activity and balanced nutrition not only support long-term metabolic health, but also promote daily well-being, energy, and emotional resilience.

It is therefore essential to underscore that daily physical exercise is not only important for reducing adipose tissue, but—more critically—for preserving and enhancing skeletal muscle mass, whose endocrine activity exerts a fundamental protective role in maintaining metabolic homeostasis [61].

### 4.4. The Health Benefit–Risk Triangle

**Figure 4:**
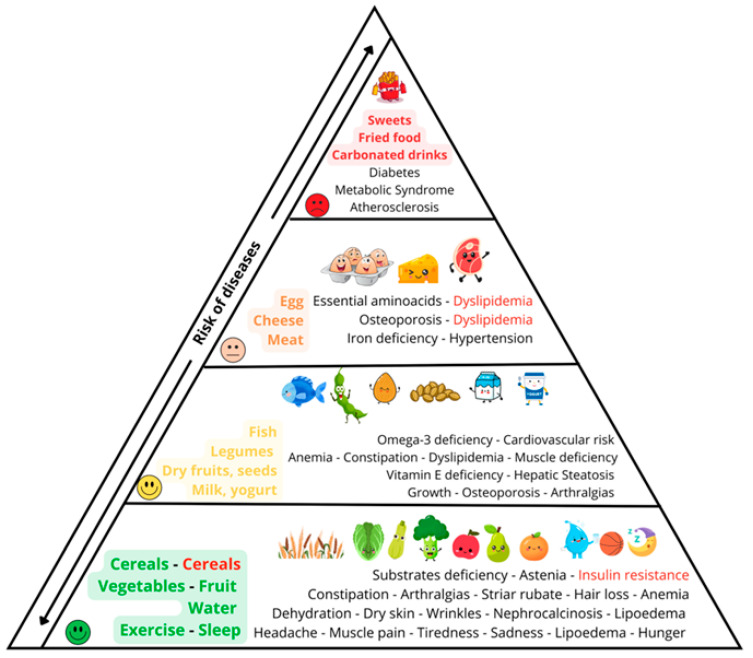
The 4th face: “The health benefit–risk triangle”.

In this triangle (Figure 4), the protective effects of a healthy lifestyle are explicitly contrasted with the pathological consequences of an unhealthy one. The figure aims to depict signs and symptoms in accessible terms even to younger children, while retaining scientific accuracy.

At the base of the triangle (depicted in green), the positive effects of regular physical activity and a normalized sleep–wake cycle are emphasized. These include prevention of headaches, myalgia, depressive mood, and excessive hunger, as well as mitigation of lipedema risk [40].

The role of adequate hydration is illustrated in relation to dry skin, wrinkling, nephrocalcinosis, and lipedema. The importance of fruit and vegetable consumption is shown with respect to skin integrity, prevention of striae rubrae, and reduction in hair loss. The scheme also considers cereal intake in its bi-model way: insufficiency may lead to substrate deficiency and inadequate energy supply, whereas excessive or improper cereal consumption is indicated as a contributor to insulin resistance and metabolic dysregulation.

In the intermediate region of the triangle (depicted in yellow), the benefits of milk and dairy products for longitudinal growth are presented. Particular attention is given to growth failure in both obese and undernourished children, for whom achieving genetic target height may not be assured. Observational evidence (Project Viva) shows that early childhood consumption of higher-fat cow’s milk was not associated with increased adiposity in adolescence and may be inversely associated with overweight/obesity risk [62]. Moreover, umbrella reviews suggest milk intake more often associates with benefits than harms for outcomes like bone health, metabolic syndrome, and cardiovascular disease [34]. Dietary calcium importance for bone health is thus supported. Nuts and seeds are noted for their high vitamin E content and a potential protective effect against hepatic steatosis. Fish intake, or the lack thereof, is portrayed in terms of omega-3 deficiency and its associated cardiovascular risk: long-chain polyunsaturated fatty acids have been shown to have protective effects in childhood cardiovascular risk profiles [63].

The upper portions of the triangle (depicted in orange and red) serve to illustrate that, while healthy nutrition plays a crucial role in protecting from disease and maintaining a good health state, its positive effects may be overridden by over-eating highly flavorful processed foods which induce metabolic and cardiovascular disease.

However, children and parents can be reminded that taste preferences are modifiable and can be influenced by repeated exposure: offering high-quality foods may help shift preferences, while highly processed foods may lose their appeal over time [64].

The visual stratification helps the patient to appreciate the degree of health risk exposure, and to understand how, over time, persistent unhealthy lifestyle factors can impair long-term health.

## 5. How to Use the Tridimensional Pyramid in an Outpatient Setting

The three-dimensional pyramid model enables clinicians, nurses, and dietitians to communicate the significance of a healthy lifestyle—comprising nutrition, eating behaviors, and physical activity—and its implications for health outcomes.

Unlike conventional pediatric guidelines, the Lifestyle Tridimensional Pyramid integrates nutrition, physical activity, and sleep into a single, visually intuitive model. It emphasizes the balance between skeletal muscle and adipose tissue rather than relying solely on BMI, and is specifically designed for interactive use with children and families to enhance understanding and engagement. Grounded in evidence from umbrella reviews, the pyramid provides a robust, multi-component framework that addresses the limitations of traditional recommendations and supports holistic, sustainable lifestyle changes in pediatric populations [45,46].

This multidimensional approach addresses limitations of previous interventions that often focus on diet or exercise alone and aligns with evidence that integrated strategies are more effective in preventing childhood obesity and associated metabolic disorders.

By presenting the standing pyramid to patients and their families, the educator indicates that the base, comprising the lower halves of the first three faces, represents the health-promoting factors. It can be readily explained that adherence to these foundational “rules” supports the development of a favorable metabolic age profile, overall well-being, and long-term good health. As one ascends the faces of the pyramid, adverse lifestyle habits become more evident, and these are depicted as accelerating metabolic ageing.

If these adverse habits continue unchecked, the pyramid metaphorically becomes unstable and may collapse. This instability serves to demonstrate to patients and families that health is severely threatened by unhealthy lifestyle choices, leading over time to metabolic and cardiovascular disease, as portrayed on the fourth face of the pyramid.

**Figure 5:**
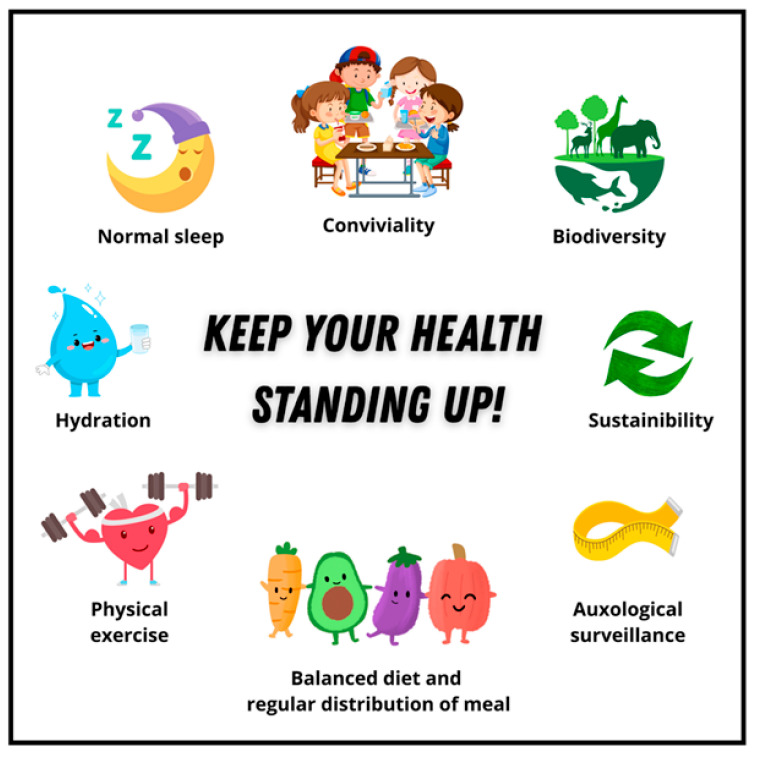
The 5th face: “Square base”.

Finally, the collapse of the pyramid, which can no longer remain stable when supported only at its apex, exposes a 5th face (Figure 5)—corresponding to the pyramid’s base—that summarizes the pillars of health that the patient must consistently uphold in daily life in order to preserve long-term health summarized with the slogan “keep your health standing up”. The “overthrowing” of the pyramid, as well as the novelty of this 3D representation, consistently enhanced understanding of the underlying health risks.

Establishing a strong and effective channel of communication between the healthcare provider and the pediatric patient is fundamental to promoting healthy behavioral change. This process requires direct, distraction-free interaction, with the food pyramid placed centrally to serve as a shared point of reference. The clinician should engage the child in a structured yet empathetic dialogue, inquiring about their daily habits, such as the frequency of vegetable consumption, breakfast routines, snack choices, and physical activity levels. Building rapport also involves tailoring the conversation to the child’s developmental stage, interests, and family context. Clinicians should demonstrate empathy toward individual challenges and social circumstances, including cultural, economic, and environmental factors that may limit access to healthy foods or safe spaces for physical activity. The Lifestyle Tridimensional Pyramid can be simplified and adapted to ensure comprehension and usability for each child and family—for example, by using age-appropriate language, visual cues, and culturally relevant examples of diet and activity. By recognizing and addressing these potential barriers, healthcare providers can help families identify practical, feasible strategies to adopt healthier behaviors within their specific circumstances, fostering sustainable lifestyle changes even in less favorable environments. Nutritional and lifestyle recommendations must be personalized: for example, in a child with highly unstructured eating habits, who refuses vegetables and consumes only sugary drinks, the initial goal should be small and achievable—such as improving hydration by gradually replacing sweetened beverages with water. Similarly, for children unable to access organized sports, it is important to convey that physical activity can be achieved through informal, everyday movement. While artificial intelligence tools may assist in providing information and suggestions, they cannot replace the human elements of trust, empathy, and personalized care that underpin the educator–child relationship [36].

## 6. Pyramid Model Fact Finding Investigation

To validate the proposed lifestyle pyramid model, a small fact-finding investigation was conducted exclusively with parents and healthcare professionals using two structured questionnaires. One questionnaire targeted parents to assess perceived interest, clarity, educational effectiveness, and the child’s awareness of healthy lifestyle behaviors. The second was administered to healthcare professionals (pediatricians and dietitians) to evaluate clarity, educational effectiveness, adherence to nutritional guidelines, and clinical applicability in outpatient settings.

The questionnaire was completed by 19 dietitians, 31 pediatricians, and 48 parents (the questionnaires are shown in Supplementary Figure S2).

Both questionnaires were presented after explaining the pyramid model, and responses were measured using a 5-point Likert scale (1 = not at all, 5 = very much). Each questionnaire included a final open-ended question: parents were asked about potential lifestyle changes they would support in their child, and healthcare professionals were invited to provide comments or suggestions. This approach allowed for a quantitative and qualitative assessment of the model’s acceptability, clarity, and perceived utility among key stakeholders.

Participation in both surveys was voluntary and anonymous. No personal identifiers or clinical data were collected, and questionnaire responses were not linked to medical records. The educational activity was delivered independently of routine clinical decision-making, and participation or non-participation had no influence on the clinical care provided.

Survey results are presented in Table 2, which reports the mean scores (on a 1–5 scale) for each evaluated parameter.

## 7. Strengths and Limitations

The main strength of this study is the innovative educational strategy based on a three-dimensional inverted pyramid model, which allows pediatric patients to visually and physically experience the consequences of unhealthy lifestyles. This experiential approach makes abstract concepts more concrete and appears to enhance understanding. Notably, families previously exposed to traditional food pyramid education reported greater appreciation of this model and a clearer grasp of the concept, particularly the meaning of the pyramid’s collapse.

The main limitation is the lack of discussion of evidence that may challenge the effectiveness of educational interventions based on nutritional models. Large-scale randomized controlled trials, such as the HEALTHY Study Group trial [65] and the Pathways randomized controlled trial [66], have shown that even comprehensive school-based programs combining nutrition and physical activity may fail to produce significant improvements in obesity-related outcomes.

This suggests that increased knowledge and engagement do not necessarily translate into measurable clinical or behavioral changes. Additional limitations include reliance on subjective feedback, absence of a control group, and lack of long-term follow-up.

## 8. Conclusions

Although recent reviews have extensively examined pediatric nutrition education and counseling strategies [53], these studies predominantly focus on school-based interventions or structured counseling programs, often addressing dietary behaviors in isolation. None of the identified frameworks integrate multiple determinants of pediatric health—including dietary habits, physical activity, sleep, and risk awareness—within a unified, child-centered, clinically applicable model. This gap underlines the need for a novel conceptual framework, such as the three-dimensional square-based food pyramid proposed in the present work. The evidence, obtained by the umbrella review and summarized in our manuscript, underpins the rationale for the Lifestyle Tridimensional Pyramid, highlighting the integration of nutrition, physical activity, sleep, and body composition in supporting pediatric obesity prevention. It is now evident that there is a pressing need to develop a large-scale tool for disseminating information on healthy lifestyles and promoting both primary and secondary prevention of obesity.

This three-dimensional pyramid enables health educators to emphasize clearly each component of a healthy lifestyle. During any given educational session, one face of the pyramid may be examined in greater detail, while always recognizing its interdependence with the entire model. With this tool, we aim to offer clinicians an accessible and innovative means of promoting health from infancy onwards.

In parallel with the widespread increase in childhood obesity, pediatric clinics are increasingly encountering normal-weight children who present with an unfavorable lean-to-fat mass ratio and laboratory findings indicative of metabolic dysfunction, such as hypertriglyceridemia and hypercholesterolemia. This underscores the fact that being within a normal weight range does not necessarily equate to being metabolically healthy. Therefore, it is essential to promote healthy lifestyle habits—including balanced nutrition and regular physical activity—not only among overweight or obese children but also among those with normal weight and their families. Adherence to a healthy lifestyle should be regarded as a universal standard of care, and appropriate health education must be provided to all pediatric patients.

The new law on obesity sets as its primary objective the establishment of a National Program for the Prevention and Treatment of Obesity, supported by a dedicated and progressively increasing allocation of public funds over the coming years. These resources will be used to finance public awareness campaigns and nutrition education in schools and among the general population, promote physical activity, and support the training of healthcare professionals.

The conducted survey enabled an initial evaluation of the model’s effectiveness among parents and yielded highly positive feedback from the healthcare professionals consulted for expert appraisal. The active involvement of families is particularly relevant: although pediatric patients should be sensitized to healthy lifestyle principles to establish the foundations for long-term health, parents play a pivotal role in health education and in ensuring adherence to healthy dietary and lifestyle behaviors during childhood. Nevertheless, future research should consider administering the questionnaire directly to pediatric patients in order to obtain first-hand data on the educational impact of the model and to further strengthen the evidence supporting its applicability.

Additional data collected in this way would be useful to validate the impact of the tridimensional pyramid in pediatric clinical practice. Also, the umbrella review summarizes lifestyle behaviors of pediatric obesity, but limitations include retrospective design, lack of prospective registration, and heterogeneity in outcomes and measurements. Evidence on the impact of integrated, multi-component interventions remains limited, with most data observational. Future research should focus on controlled studies evaluating the Lifestyle Tridimensional Pyramid, assessing feasibility, adherence, behavioral change, and metabolic outcomes. Standardized assessment of diet, physical activity, sleep, and body composition will strengthen comparability and support practical application in pediatric settings.

We firmly believe that the tool we propose will prove to be extremely useful, as it will support the launch of public information campaigns, targeted training for healthcare professionals (including general practitioners, pediatricians, and National Health Service personnel), and a variety of school-based, community, and local-level initiatives. These efforts aim to prevent obesity, raise public awareness, and promote healthy lifestyles.

## Figures and Tables

**Table 2:** “Investigation results”.

Family Evaluation Parameters	Parents	
Appreciation	100% evaluation: 5/5	
Clarity	96% evaluation: 5/5 + 4% evaluation: 4/5	
Awareness	98% evaluation: 5/5 + 2% evaluation: 4/5	
Comments and Lifestyle changes	- Positive parental feedback on nutrition education - Physical activity at least on weekends, - Regular breakfast, - Reduced screen time before sleep, - No sugar-sweetened beverages, - Vegetables twice daily	
Professional evaluation	Dietitians	Pediatricians
Effectiveness	100% evaluation: 5/5	100% evaluation: 5/5
Alignment with clinical guidelines	96% evaluation: 5/5 4% evaluation: 4/5	96% evaluation: 5/5 4% evaluation: 4/5
Applicability in outpatient settings	98% evaluation: 5/5 2% evaluation: 4/5	100% evaluation: 5/5
Comments	Innovative explanation for children	Improved patient attention

## Data Availability

No new data was created.

## Supplementary Materials

The following supporting information can be downloaded at: https://www.mdpi.com/article/10.3390/nu18081209/s1, Figure S1: “PRISMA 2020 flow diagram for umbrella review; Figure S2A: Pyramid evaluation questionnaire for healthcare professionals; Figure S2B: Parents’ questionnaire—perception of nutrition education and lifestyle.
