# Supplementary material for: How to Teach Healthy Life-Style Efficiently in a Pediatric Outpatient Setting: Proposal of an Innovative Tridimensional Pyramid
Source: Nutrients. 2026 Apr 11;18(8):1209. doi: 10.3390/nu18081209 (PMC13119281; doi:10.3390/nu18081209)
Supplement: Supplementary file 1 [file nutrients-18-01209-s001.zip › nutrients-4179253-supplementary.pdf]

Supplementary Figure S1

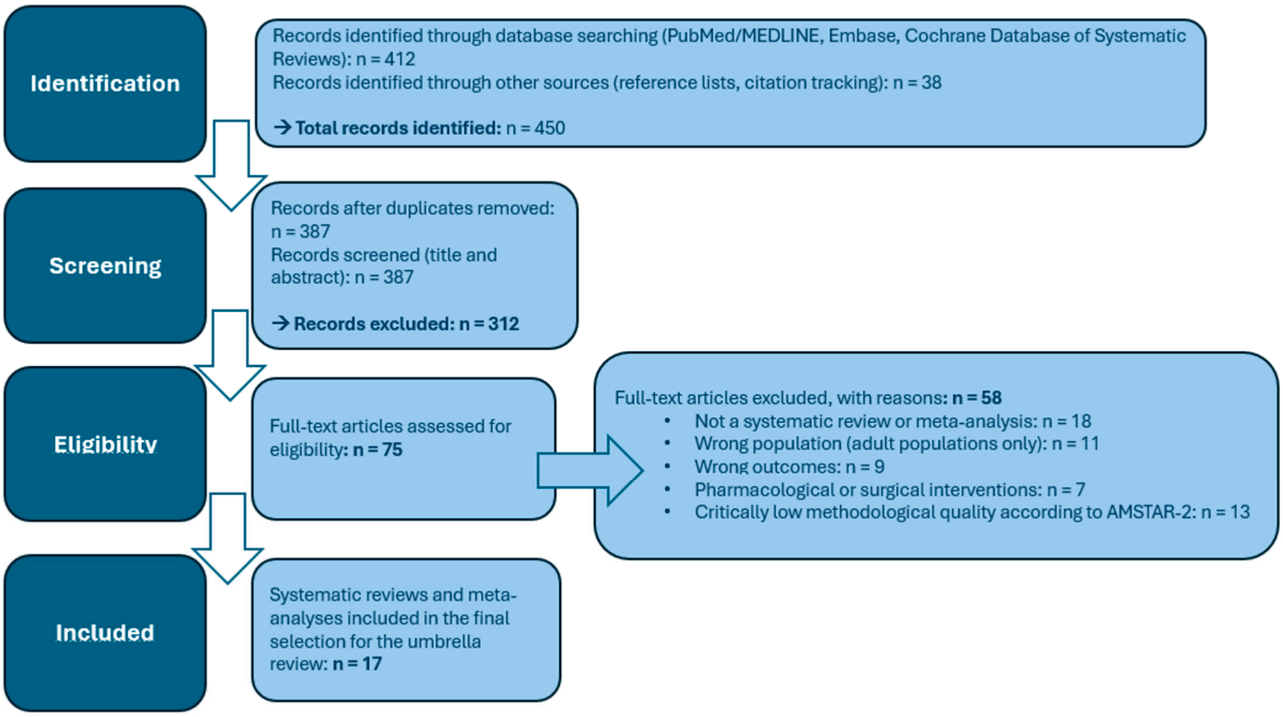

PRISMA 2020 flow diagram for umbrella review

PYRAMID EVALUATION QUESTIONNAIRE FOR HEALTHCARE PROFESSIONALS (Figure S2A)

Section	Question	1–5 Scale
Clarity and Comprehension	1. The food pyramid model is clear and easy to understand.	1 2 3 4 5
	2. The information and guidance provided are presented in a coherent and logical manner.	1 2 3 4 5
Educational Effectiveness	3. The model can facilitate communication with pediatric patients regarding balanced nutrition.	1 2 3 4 5
	4. I believe that using the model can improve children's understanding of nutritional concepts.	1 2 3 4 5
Adherence to Guidelines	5. The model is consistent with national/international nutritional guidelines.	1 2 3 4 5
	6. The model reflects current scientific recommendations in pediatric nutrition.	1 2 3 4 5
Clinical Applicability	7. I believe the model can be easily used in outpatient settings during pediatric visits.	1 2 3 4 5
	8. The tool is practical and realistic to integrate into daily clinical practice.	1 2 3 4 5
Comments and Suggestions	9. Which aspects of the model do you find most effective?	–
	10. What modifications would you suggest to improve its clarity, effectiveness, or applicability?	–

___

___

___

PARENTS' QUESTIONNAIRE – PERCEPTION OF NUTRITION EDUCATION AND LIFESTYLE (Figure S2B)

1. General Information

• **Child's age: ___ years**
• **Child's sex: ☐ M ☐ F**

2. Evaluation of the Educational Activity

Please indicate how much you agree with each statement using a scale from 1 to 5:
(1 = Not at all / 5 = Very much)

1. **The explanation was engaging and captured my child's attention.**
   1☐ 2☐ 3☐ 4☐ 5☐

2. **The information was explained in a clear and understandable way.**
   1☐ 2☐ 3☐ 4☐ 5☐

3. **The proposed pyramid model helped my child better understand how to take care of their health.**
   1☐ 2☐ 3☐ 4☐ 5☐

4. **After the explanation, my child seems more aware of their food choices and lifestyle habits.**
   1☐ 2☐ 3☐ 4☐ 5☐

5. **Do you think anything will change in your child's lifestyle or eating habits? If yes, what?**

____
____
____
